# *In vitro* histological evaluation of the surgical margins made by different laser wavelengths in tongue tissues

**DOI:** 10.4317/jced.52830

**Published:** 2016-10-01

**Authors:** Ana-Salvaterra Azevedo, Luís-Silva Monteiro, Fernando Ferreira, Maria-Leonor Delgado, Fernanda Garcês, Sofia Carreira, Marco Martins, Juan Suarez-Quintanilla

**Affiliations:** 1 Stomatology Department, Valongo Unit - São João Hospital Centre, Porto, Portugal; 2 Morphology Department, University Institute of Health Sciences, Paredes, Portugal; 3 Medicine and Oral Surgery Department, University Institute of Health Sciences, Paredes, Portugal; 4 Pathology Department, University Institute of Health Sciences, Paredes, Portugal; 5 Physiology Department, University Institute of Health Sciences, Paredes, Portugal; 6 Medical Morphology Department, University of Santiago de Compostela, Coruña, Spain

## Abstract

**Background:**

Lasers have become standard tools for the surgical treatment of oral lesions. The purpose of this study is to determine the surgical margins and histologically evaluate the tissue thermal effects induced by different types of surgical instruments.

**Material and Methods:**

Cuts were made in pork tongues’ mucosa with different lasers (Er:YAG at 2W with and without air / water spray and at 4W with and without air / water spray; CO2 at 3.5W and 7W in pulsed mode and at 7W in continuous mode; the diode laser at 3.5W and boost 3.5W in pulsed mode; Nd:YAG at 6W, 40Hz and electroscalpel at 5W and conventional scalpel as control. Macroscopic and microscopic morphological changes were evaluated.

**Results:**

The results of this study showed that the surgical instruments that caused greater tissue damage extension were: the Nd:YAG laser (670.68μm), the diode 3.5W and boost PW (626.82μm), the CO2 7W CW (571.18μm), the CO2 at 7W PW (485.45μm), the diode 3.5W PW (456.15μm), the electroscalpel (409.57μm) and lastly the CO2 laser 3.5W PW (306.19μm) and Er:YAG (74.66μm) laser, regardless of power, mode or air / water spray used. An association between the Tissue Damage Extension and the Degree of Carbonization (r = 0.789; *P* = 0.01), and an association between the Tissue Damage Extension and Regularity of the Incision were found (r = -, 299; *P* = 0.01).

**Conclusions:**

The results of this study suggest that lasers can be used in soft tissues biopsies of the oral cavity, enabling a correct histopathological analysis, as long as the biological effects of each laser type are considered. The Er:YAG laser revealed its potential for biopsies of the oral mucosa ensuring a successful histological evaluation and the CO2 laser at 3,5W in pulsed mode presented itself as the best choice for surgeries with hemostasis.

** Key words:**CO2 laser, diode laser, Er:YAG laser, laser surgery, Nd:YAG laser, oral mucosa, thermal effect.

## Introduction

Lasers have become standard tools for the surgical treatment of oral lesions. The use of laser technology in the surgical treatment of oral lesions aims to provide benefits to both the surgeon and the patient (1).

The acquired clinical experience over the past decades ensures a number of advantages in the use of laser versus scalpel during soft tissue surgery, including a high degree of decontamination of the surgical field, minimal postoperative bleeding and a significant decrease in pain and postoperative inflammation (1-4).

During the application of laser in oral soft tissues, the light energy is transformed into thermic energy that turns into heat on the target tissue to produce the wanted effect (5). This photothermal effect can produce changes in the tissues, and if the soft tissues are to be examined by an optical microscope, artifacts can make the histopathological interpretation difficult. Therefore the reduction of peri-incisional damage is crucial in oral pathology (6,7).

Different types of laser have shown utility and efficiency in dentistry, including CO2, Er:YAG, diode or Nd:YAG lasers (8). The CO2 laser, due to its affinity with water, has become a highly used instrument in the treatment of oral mucosa lesions by oral surgeons (9). Its penetration is poor, which makes the CO2 laser particularly suited for being used close to critical anatomical structures (10-12). The CO2 laser is an ideal tool for a clean bloodless surgical field because of its hemostatic capacity in vessels with less than 0.5 mm diameter (8). For the treatment of vascular lesions in vessels with more than 7-mm diameter, like oral hemangiomas, some authors (13) advocate the Nd:YAG or diode laser. Nevertheless, the strong coagulation effect can lead to artifacts that may influence the histological diagnosis (1).

The Er:YAG laser promotes rapid healing due to the short side thermal effect it generates (10,14). However, its drawback is that the interventions won’t be so hemostastic as the ones using the CO2, Nd:YAG or diode laser (10).

The diode and the Nd:YAG lasers are less absorbed by water and more absorbed by hemoglobin and melanin thus having a deeper effect on tissues (15). Nevertheless, in general dentistry it is now a widely accepted treatment aid, with a broad range of applications in oral soft tissue surgery (16).

There are only a few studies that have systematically analysed atypical cytological or structural changes in oral epithelium, or its association with different lasers and power (17). Most of the described cases have used the extent of the hyalinised tissue or coagulated tissue adjacent to the irradiated margins to measure the results, and only occasionally were cytological artifacts in the incision considered. Few authors described the type of laser considered suitable for soft tissue biopsy (5).

The purpose of this study is to determine the macroscopic and microscopic morphological changes in the surgical margins in tongue tissue (*ex vivo*) induced by different surgical instruments, including various types of laser.

## Material and Methods

-Sample

For the purpose of this *ex vivo* study, 10 pig cadavers’ tongues were used, 24 hours after slaughter. The total sample consisted of 120 incisions made with Er:YAG laser (N = 40, 33.3%), CO2 laser (N = 30, 25%), diode laser (N = 20, 16.7% ), Nd:YAG laser (N = 10, 8.3%), electroscalpel (N = 10, 8.3%), and cold scalpel (N = 10, 8.3%).

-Evaluation Tools

The emission for each laser parameters used were those recommended by the manufacturer for soft tissue surgery, and some other variants were selected by the researchers for the purpose of the study. Each surgical instrument used, and its respective parameters, correspond to each tongue incision: CO2 laser by DEKA® Smart US-20D with a wavelength of 10,6μm was used with a no-contact handpiece for three different types of application: 3.5W in pulsed mode (PW) at 50Hz, 7W PW at 50Hz and 7W in continuous mode (CW). Another laser used was the Nd:YAG by DEKA® Smart A10 with a wavelength of 1.06μm using fiber of 300 μm, 6W power with contact mode and frequency of 40Hz. The Er:YAG laser by DEKA® Smart 2940 D plus with a wave-length of 2,940μm was used with a no-contact piece for four different types of applications: 2W 10Hz and 0.2J short pulse with air / water spray, 2W at 10 Hz and 0.2J short pulse without air / water spray, 4W 10Hz and short pulse 0.4J with air / water spray and 4W 10Hz and 0.4J short pulse without air / water spray. The diode laser of LITEMEDICS® with a wavelength of 980nm was used in contact mode for two different applications: 3.5W and 3.5W Boost PW. It was also used a Servotome electroscalpel by SATELEC® at 5W of power, and for the specimens control a scalpel blade number 15 by KIATO® was used.

-Data Collection Procedures

• Surgical procedure

The samples were stored at 2-4ºC during transportation and 100% humidity to prevent tissue degradation as reported in the literature (5,18).

The surgical technique was achieved by directing the laser beam perpendicularly to the dorse of the tongue. Samples were collected by the same dentist to prevent errors from interindividual differences. A second operator then placed the samples in sterile containers with formalin buffered at 10%. The samples were sectioned with a minimum margin of 10mm from the study cut.

• Macroscopic evaluation

Based on the criteria of Cercadillo-Ibarguren *et al.* (5) with respect to the tissue carbonisation, we proceeded to the macroscopic evaluation of the incision based on a scale of 0 to 4, in which 0 corresponds to no color detected in the incision, 1 corresponds to a brownish color on the surface of the incision, 2 when brown is detected deep into the edges of the incision, 3 to classify a black color on the surface of the incision and 4 to black in depth.

• Histologic evaluation

The specimens were fixed, dehydrated and embedded in paraffin. Serial sections were performed with 3µm thickness. They were conventionally stained with haematoxylin-eosin (HE) and were also dyed with Masson Tricrome (TM) to control false positives. Overall, we obtained 240 histological preparations (120 HE and 120 with TM), and they were evaluated on a ZEISS Axio®optical microscope with Axiovision® software (release 4.6.3).

The histological variables assessed at specimens’ level are based on the criteria established by Vescovi *et al.* (2). Epithelial changes in the core include core, cytoplasmic and membrane modifications, and possible loss of intraepithelial and subepithelial adhesion; modification of connective tissue including charring and desiccation; morphology and regularity of the incision on a scale of 0 to 4 in which they were classified as “regular” (≥2) when it presents a smooth, linear border mostly of incisional margin, and as “irregular” (<2) in the presence of a rough and uneven edge in most of the incision, where level 4 represents the highest quality and 0 the worst incisional quality; Extent of Thermal Tissue Damage (ETTD) expressed in microns by measuring the greatest distance from the edge of the incision to the end of the laser thermal damage in the tissue. At the same time, a photographic file was compiled. The samples were coded and a double blind analysis for each type of laser setup used was made by two pathologists to reach a consensus for each case.

-Analysis procedure data

The data analysis was obtained by descriptive and inferential statistics, using the SPSS-22.0 software (Statistical Package for Social Sciences).

Given that the null hypothesis (H0) to the Kolmogorov-Smirnov normality test is that data is normally distributed, and as the result of *P*-value was (*P* < 0.05) for the variables under study, we reject the null hypothesis (H0) and we assume that the sample does not follow a normal distribution in the variables under study. Thus, non-parametric tests like Spearman correlation test, Mann-Whitney test, Kruskal-Wallis test and the Chi-Square test were used.

## Results

-Macroscopic evaluation

With the exception of the Er:YAG laser with mean values of 0 and 1, all the other surgical instruments showed a significant charring average value as can be seen in  Table 1. The Nd:YAG laser and CO2 laser at 7W CW caused greater tissue carbonization with average values of 4.

**Table 1:**
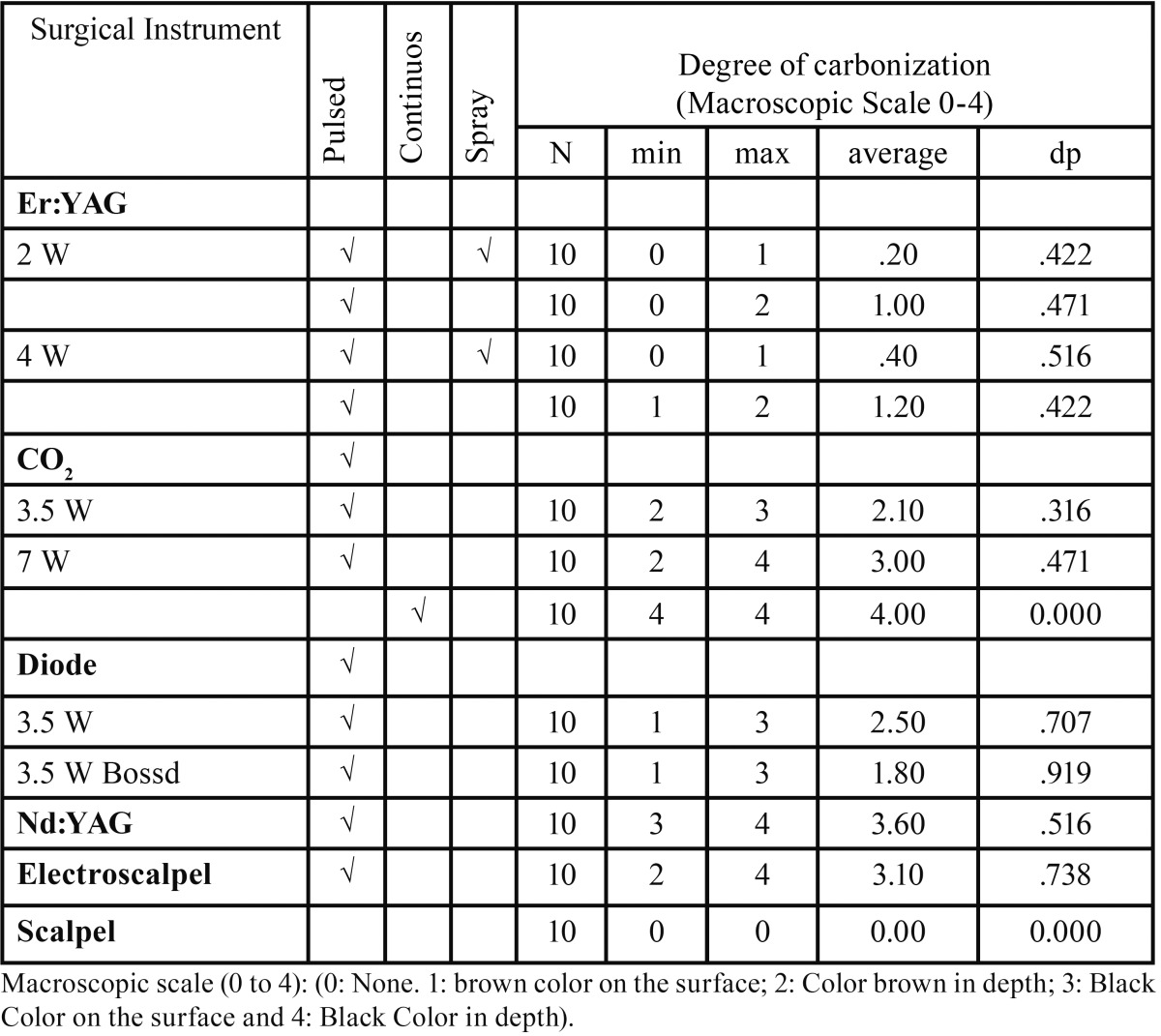
Degree of carbonization by instrument, power, mode and spray.

An association was found between the ETTD and Carbonization Degree which indicates a strong positive significant correlation (r= 0,789; *P* = 0.01).

-Histological evaluation

**Figure 1:**
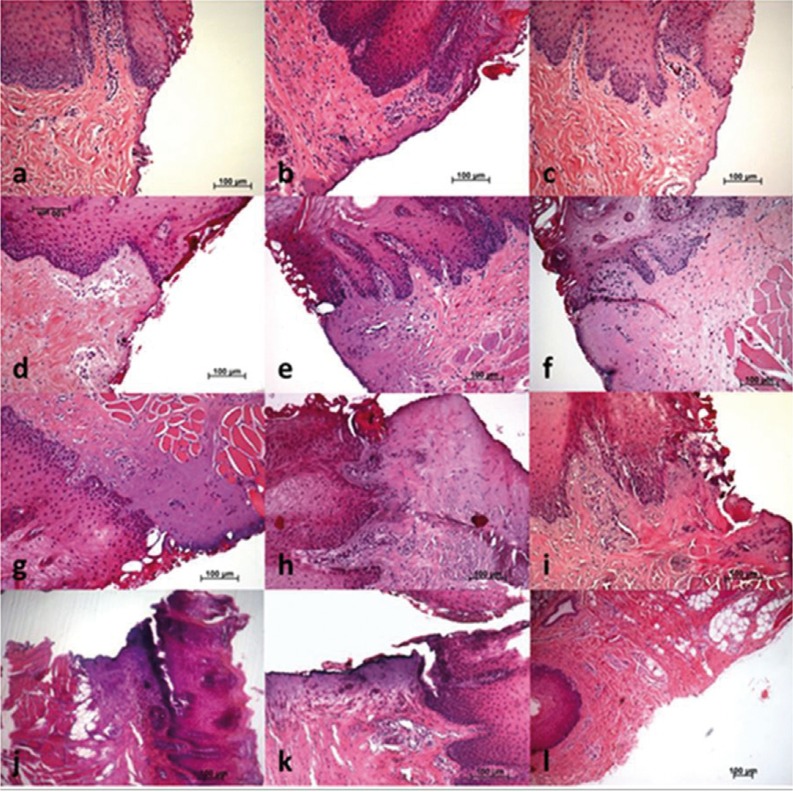
Incisional margin of the tongue tissue samples (haematoxylin and eosin-staining): a) Er:YAG Laser at 2W PW with air / water spray (x100 magnification); b) Er:YAG Laser at 2W PW without air / water spray (x100 magnification); c) Er:YAG Laser at 4W PW with air / water spray (x100 magnification); d) Er:YAG Laser at 4W PW without air / water spray (x100 magnification); e) CO2 Laser at 3,5W PW (x100 magnification); f) CO2 Laser at 7W PW (x100 magnification); g) CO2 Laser at 7W CW (x50 magnification); h) Diode Laser at 3,5W PW (x100 magnification); i) Diode Laser Boost at 3,5W to (x100 magnification); j) Nd:YAG Laser (x50 magnification); k) Electroscalpel (x100 magnification); l) Cold Scalpel (x50 magnification).

**Figure 2:**
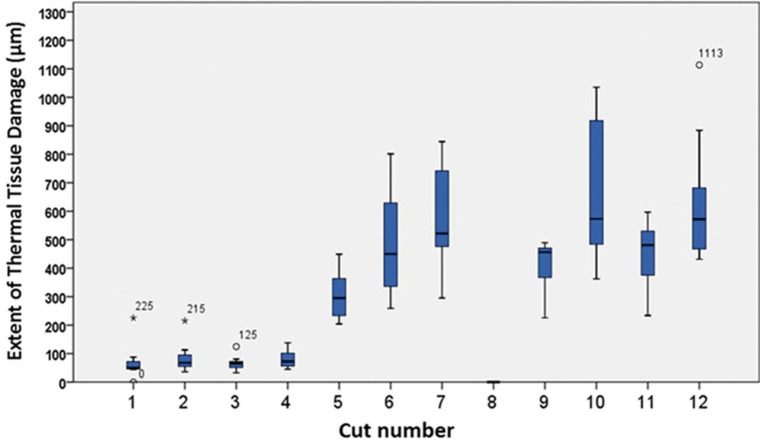
Box plot of tissue damage extension (µm) of instruments surgical used. by power mode and air / water spray. Cut 1: Er:YAG 2W with spray. Cut 2: Er:YAG 2W without spray. Cut 3: Er:YAG 4W with spray. Cut 4: Er:YAG 4W without spray. Cut 5: CO2 3.5W pW. Cut 6: CO2 7W pW. Cut 7: CO2 7W cW. Cut 8: Scaplel. Cut 9: Electroscalpel. Cut 10: Nd:YAG. Cut 11: Diode 3.5W pW. Cut 12: Diode Boost pW.

The values of tissue changes by type of artifact (nuclear, cytoplasmic or connective) versus surgical instrument, power, spray and mode used are shown in  Table 2. The instruments with the highest number of tissue changes were the electroscalpel and Nd:YAG laser, and the one with fewer changes, particularly at epithelial level, was the Er:YAG laser, regardless of power, mode or spray used (Fig. 1). Significant differences were found in ETTD between the number of tissue changes in Score Nuclei (*P* < 0.001); Score Cytoplasm (*P* < 0.001) and Connective Score (*P* < 0.001). It was observed a higher ETTD in the presence of a greater number of changes within each score. Although not statistically significant, an association between the type of surgical instrument and its power and the tissue changes (instrument and power Vs picnotic Core / core spindle / core hyperchromatic / cytoplasmic hyperchromatism / cell fusion (*P* < 0.001); instruments and power vs adherence loss (*P* = 0.02); instruments and power vs carbonization (*P* = 0.035), except for change “dissection” (*P* = 0.214)) was found. It was also possible to behold statistically significant differences (*P* <0.001) in ETTD related to the types of surgical instruments used. The values of ETTD for surgical instrument, power, mode and air / water spray are shown in  Table 3 and (Fig. 2). The instrument with the highest ETTD was the Nd:YAG laser (670.68μm), then come the diode laser at 3.5W Boost PW (626.82μm), the CO2 laser at 7W CW (571.18μm), the CO2 laser at 7W PW (485.45μm), the diode laser at 3,5W PW (456.15μm), the electroscalpel (409.57μm) and lastly the CO2 lasers at 3,5W PW (306.19μm) and Er:YAG laser (74.66μm) regardless of their power, mode or air / water spray (Fig. 1). It was found that there is an average lower ETTD in the presence of air / water spray and a higher average in the absence of air / water spray of the Er:YAG laser, although the difference was not statistically significant (*P* = 0.123). As expected, the scalpel control specimens demonstrated no thermal damage at the margins of the incision (Fig. 1l).  Table 4 shows the values of the regularity of the incision for surgical instrument, power, mode and air / water spray. The most regular incision was obtained with the CO2 laser at 3,5W in pulsed mode and the less regular incision with the Nd:YAG laser. An association between ETTD and the regularity of the incision was found (r = -, 299; *P* = 0.01).

**Table 2:**
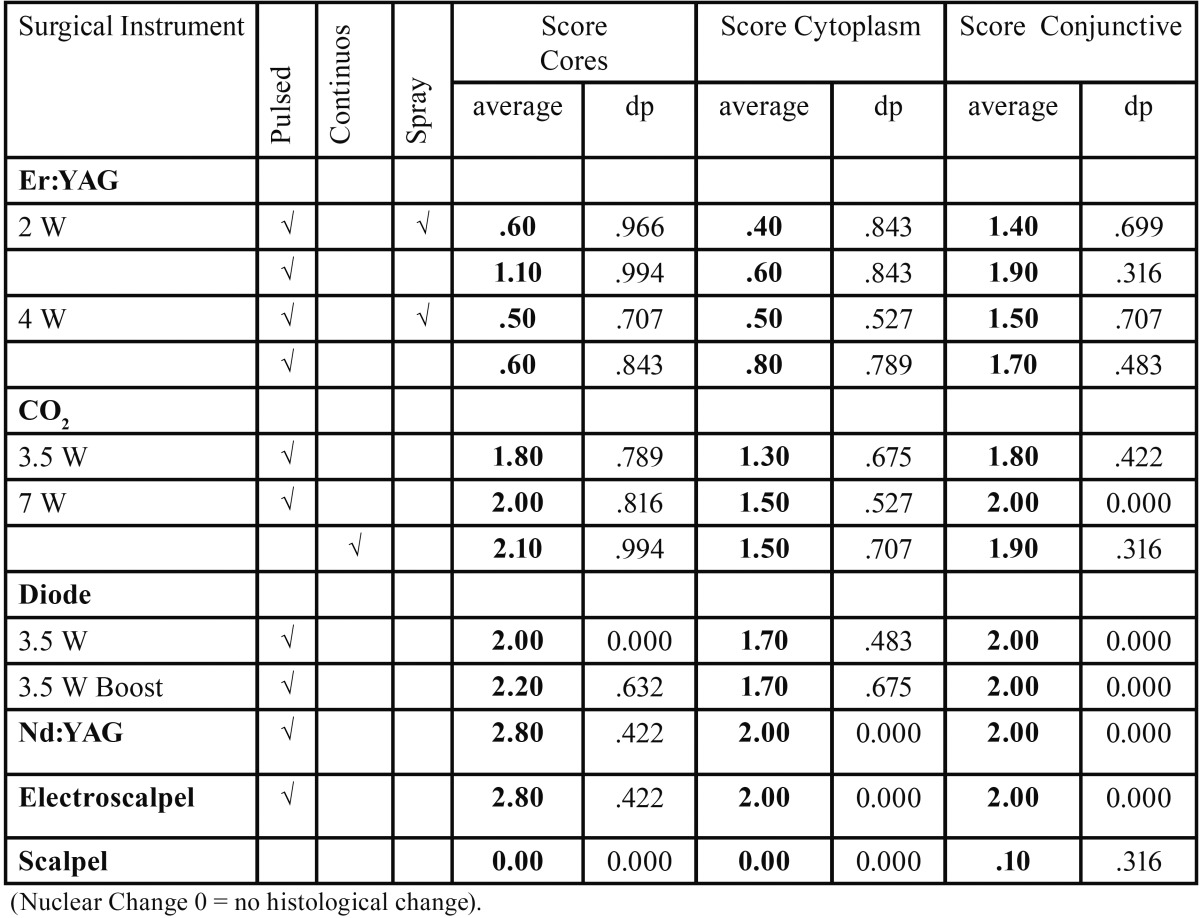
Nuclear, cytoplasmic and connective changes for Surgical Instrument, Power, Mode and Spray.

**Table 3:**
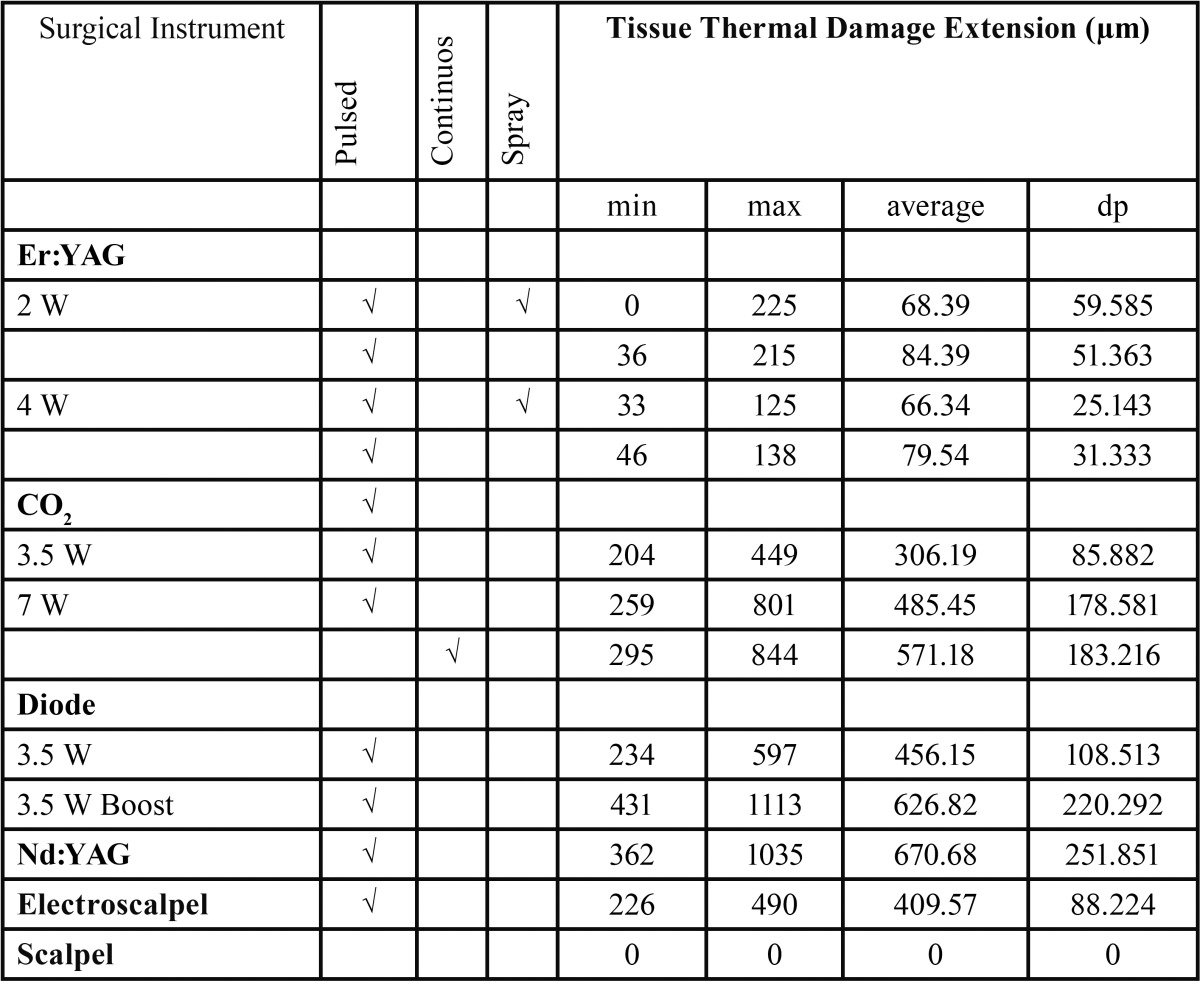
Tissue Thermal Damage Extension by type of surgical instrument, power, mode and (spray air / water).

**Table 4:**
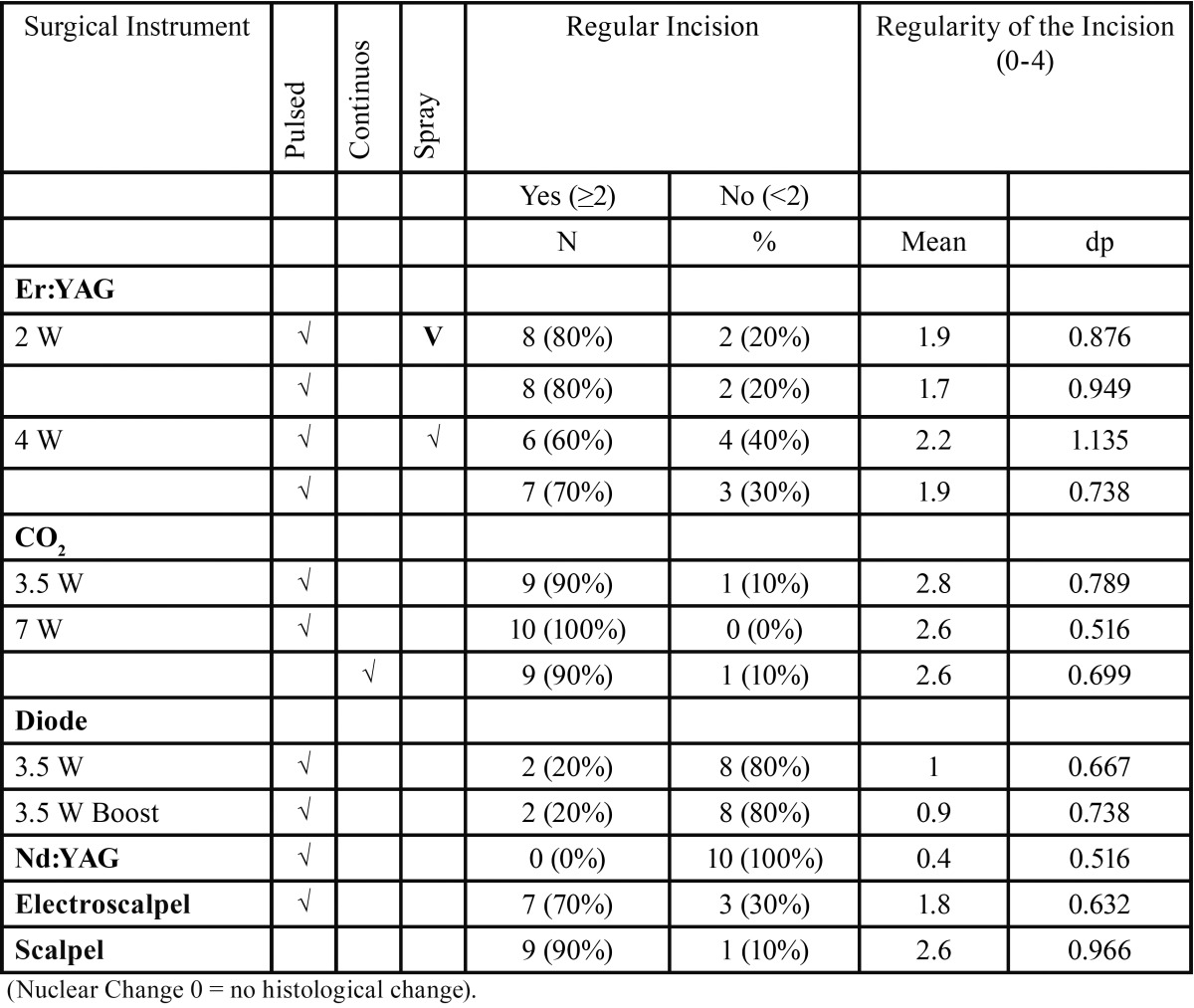
Nuclear, cytoplasmic and connective changes for Surgical Instrument, Power, Mode and Spray.

## Discussion

There are several studies on the use of laser in oral biopsy of soft tissue, but only some of them focus on the damage caused by this device in peri-incisional margins of tissue samples, and only a few include associated variables, as the power, wavelength or emission mode.

Recent studies (15,18-20) have shown that the CO2, Er:YAG, Nd:YAG and diode lasers proved to be ideal devices for oral soft tissue with little thermal damage, allowing a correct histological diagnosis. We have showed in this paper that the artifacts caused by different types of laser are limited to a small area of tissue and don’t affect the entire fragment.

The instruments with the highest degree of carbonization of the tissue were the Nd:YAG and CO2 7W CW lasers, and the one with the lowest charring was the Er:YAG laser. The paper by Cercadillo-Ibarguren *et al.* (5) regarding macroscopic classification, states that the CO2 and diode lasers always produced higher values of carbonization, probably due to higher power level, continuous mode and because of the wavelength used in their research (830nm).

The present study found a significant association between ETTD and carbonization degree, showing that the higher the degree of charring caused by the surgical instrument, the higher the ETTD induced on the specimen. In this case the Nd:YAG and CO2 7W CW lasers with grade 4 carbonization also had the highest ETTD values. While the Er:YAG laser with the lowest values of thermal damage caused a non-relevant carbonization without air / water spray and no carbonization at all with air / water spray regardless of the power used. These results seem to indicate that in soft tissue surgery of the oral mucosa, the carbonization degree may be a clinical indicator of the damage that is being induced in the tissue.

Concerning the regularity of the incision, the best result was obtained with the CO2 laser, regardless of mode or power, and the worst result with the Nd:YAG laser. These results confirm the ones in the study of Merigo *et al.* (18), who reported good and regular results with the CO2 laser but weaker ones with the Nd:YAG laser. The intermediate quality of the incisions with the Er:YAG laser are concurrent with the results of these authors and do not seem to be related to the presence of air / water spray.

When we compared the incision regularity with ETTD, we observed that more regular cuts of the incision corresponded to cases with lower ETTD. The results of Vescovi et al. (1) support this hypothesis in human oral mucosa studies with Nd:YAG laser at 3.5W and 5W, in which they didn’t find a statistically significant difference, although the incision was better and ETTD lower in the specimens obtained with lower power laser.

All types of surgical instruments used in this study induced the same Tissue Artefact Changes, mainly located in the basal and suprabasal layers of the lingual epithelium, according to many authors (17,21), and its connective tissue. The surgical instruments that induced the higher number of artifacts were the Nd:YAG laser and electroscalpel mainly at a nuclear level; the Er:YAG laser generated fewer changes at the epithelial level (nucleus and cytoplasm) as described by Merigo *et al.* (18). However, loss of adherence was higher with the electroscalpel, the diode laser at 3.5W boost and the Nd:YAG laser, while Merigo *et al.* (18) only found this in the last laser referred.

The electroscalpel produced similar values of tissue changes to the ones obtained with the Nd:YAG laser, which is in line with the comparative study of laser surgery and electrosurgery by Vitale *et al.* (22) that found the greater damage in electrosurgery biopsies particularly at the epithelial level.

In the ETTD analysis by histological artefact induced by the different surgical instruments, it can be stated that the measure of thermal damage was normally higher in the presence of these tissue changes, suggesting that in surgical margins with higher ETTD, more histological artefacts will be found. This difference in ETTD between the presence and absence of these tissue changes was statistically significant. And while this is an expected result, it strengthens the quality of the sample under study. The results of Vescovi *et al.* (1) with Nd:YAG laser, although not statistically significant, were parallel, meaning that a higher power laser induced higher epithelial, conjunctival and vascular changes, coinciding with a higher thermal tissue damage. The surgical instrument that showed lower ETTD was the Er:YAG laser followed by the CO2 laser at 3.5W in pulsed mode; the laser that induced higher thermal damage was the Nd:YAG laser. This result is consistent with the *ex vivo* study by Merigo *et al.* (18) at different wavelengths.

Er:YAG laser achieved the best performance in terms of histological anatomy, and the lowest marginal thermal damage highlighting the power of 2W with air / water spray. In view of these results, although not statistically significant, it can be stated that in this study the presence of the spray minimized the risk of thermal damage without charring effects. So we are in line with Zaffe *et al.* (19), Merigo *et al.* (18) and Romeo *et al.* (6) as the best results in terms of “respect for the tissue” were obtained by the Er:YAG laser. However Tamarit-Borràs *et al.* (10) consider this laser to have a lower utility in soft tissue, because it doesn’t offer good hemostasis during surgery.

Though relevant to CO2 and diode lasers, tissue changes were much more evident with the Nd:YAG laser, maybe because of the warming effect of the tissues and its deep absorption, compared with other wavelengths (23), its light is primarily absorbed by hemoglobin and melanin allowing a deep penetration of energy in the tissue. The Nd:YAG laser proved to be the more aggressive surgical instrument, having exceeded up to 1 mm in one of its incisions; Romeo *et al.* (24) had already described from severe damage to extensive detachment of at least 1.5 mm when testing the effect of different lasers in pig tongues. In fact, in the work of Merigo *et al.* (18), the temperature increase in depth was most consistent with the diode and Nd:YAG lasers, which can definitely be related to the extent of tissue change. Vescovi *et al.* (1) underwent a preliminary histological analysis of human oral mucosa samples, comparing the Nd:YAG laser with traditional scalpel and concluded that this laser induces serious thermal effects in small samples (less than seven millimeters) regardless of frequency and power used.

A bibliographical analysis will reveal that the CO2 laser is in fact one of the most useful instruments for soft tissue surgery especially concerning human lesions, because of important advantages, like the hemostasis capacity, and the vast experience that surgeons have with this laser (9,10). Seoane *et al.* (21) concluded that the CO2 laser (3W-12W) generates thermal epithelial damage not necessarily related to the power employed. However, in our study, ETTD induced by this laser at 3.5W seems to be the most suitable for the preservation of tissues, with thermal injuries of, on average, 306.19µm, whilst the 7W PW laser produced, on average, 485.45 µm, and the CO2 laser at 7W CW caused greater peripheral thermal damage, with extended dermoepithelial detachment and homogenization of the chorion, damaging, on average, 571.18 µm; nevertheless, all below 1 mm extension. However, this difference between the continuous and pulsed mode of the CO2 laser was not statistically significant. Indeed, Suter *et al.* (11) indicate that both laser modes are suitable for biopsies of the oral cavity. In our study, ETTD with CO2 laser obtained an average value of 454.27µm, with a maximum value of 844,37μm, while other results reported range from 70 to 750μm (17,18,21,25), which can justify the need to include an additional amount of adjacent healthy tissue that exceeds the expected extent of epithelial thermal damage.

The thermal effect of the diode laser in this study was wide, and induced a lower ETTD average at 3.5W than that generated at 3.5W boost. Other authors reported smaller thermal effects, from 321,4μm (26) to 623 μm (15), but a lower power was used in both cases.

Values that are close to the ones in our study, up to 750μm with 3W and 5W power, were reported (18), but using a wavelength of 808nm. Romeo *et al.* (24) found differences in the thermal effects of the laser diode of 980nm and 808nm in a pig’s tongue, with the longer wavelength achieving an extensive general thermal effect; the chorion was corrupted by more than 1.5mm and the epithelium by more than 1 mm with a wide dermoepithelial detachment. With the diode laser at 808nm in pulsed mode, the peri-incisional cell damage was evidently reduced, showing the best results, with a peripheral damage of less than 1mm.

Histological evaluation of the specimens revealed a markedly longer ETTD in the group of the incisions with CO2 at 7W, Nd:YAG and diode lasers compared to the electroscalpel group. This observation has been recognised in some studies (27,28) but has also been contradicted by others (29,30).

ETTD induced by surgical instruments was observed, from the highest to the lowest result in: the Er:YAG laser, the CO2 laser at 3.5W PW, the electroscalpel, the diode laser at 3.5W PW, the CO2 laser at 7W PW, the CO2 laser at 7W CW, the diode laser at 3.5W Boost PW and finally the Nd:YAG laser.

The small number of tissue changes and lower ETTD induced by Er:YAG laser appears to be an indicator of its potential for soft tissue surgery of the oral mucosa ensuring a successful histological evaluation. However, because it doesn’t provide effective hemostatic properties, the laser which indices less tissue damages and has a superior hemostasis capacity is the CO2 laser at 3.5W PW. Moreover, this laser was the instrument that offered the most regular incisions. The Nd:YAG lasers obtained the worst results in the preservation of peri-incisional tissue, reinforcing the need of an adequate knowledge of its characteristics and appropriate choice of the parameters associated to a training period.

As conclusion, our results show that lasers may be used in soft tissue surgery of the oral cavity, as long as the biological effects related to the use of each type of laser are understood and respected. The Er:YAG laser may be the laser of choice for biopsies of the oral mucosa because of the minimum histological artefacts observed in this paper, ensuring a valid histological evaluation, followed by the CO2 laser at 3.5W in pulsed mode, especially when the surgeon needs more hemostasis on the surgical field.
